# Is epileptiform activity related to developmental language disorder? Findings from the HelSLI study

**DOI:** 10.1016/j.cnp.2023.03.004

**Published:** 2023-04-11

**Authors:** Hanna-Reetta Lajunen, Marja Laasonen, Pekka Lahti-Nuuttila, Miika Leminen, Sini Smolander, Sari Kunnari, Eva Arkkila, Leena Lauronen

**Affiliations:** a Department of Clinical Neurophysiology, University of Helsinki and Helsinki University Hospital, Finland; b Department of Otorhinolaryngology and Phoniatrics, Head and Neck Surgery, Helsinki University Hospital and University of Helsinki, Helsinki, Finland; c Logopedics, School of Humanities, Philosophical Faculty, University of Eastern Finland, Joensuu, Finland; d Department of Psychology and Logopedics, Faculty of Medicine, University of Helsinki, Helsinki, Finland; e Unit of Analytics and Data Services, Helsinki University Hospital, Helsinki, Finland; f Research Unit of Logopedics, Faculty of Humanities, University of Oulu, Finland; g Department of Clinical Neurophysiology, University of Helsinki and Helsinki University Hospital and Epilepsia Helsinki, Finland

**Keywords:** EEG, Interictal epileptiform discharge (IED), Spike, Developmental language disorder (DLD), Smoking, Pregnancy

## Abstract

**Objective:**

To study if interictal epileptiform discharges (IEDs) are associated with language performance or pre-/perinatal factors in children with developmental language disorder (DLD).

**Methods:**

We recorded routine EEG in wake and sleep in 205 children aged 2.9–7.1 years with DLD, without neurologic diseases or intellectual disability. We examined the language performance of the children and collected data on pre-/perinatal factors.

**Results:**

Interictal epileptiform discharges were not associated with lower language performance. Children with so-called “rolandic”, *i.e*. centrotemporoparietal, IEDs had better language skills, but age explained this association. Most pre-/perinatal factors evaluated did not increase the risk of rolandic IEDs, except for maternal smoking (OR 4.4, 95% CI 1.4–14). We did not find electrical status epilepticus during slow-wave sleep (ESES)/spike-and-wave activation in sleep (SWAS) in any children.

**Conclusions:**

Interictal epileptiform discharges are not associated with lower language performance, and ESES/SWAS is not common in children with DLD.

**Significance:**

Routine EEGs do not bring additional information about language performance in children with DLD who do not have any neurologic diseases, seizures, intellectual disability, or regression of language development.

## Highlights

• EEG findings not related to language skills in developmental language disorder.
• Electrical status epilepticus in sleep not found in developmental language disorder.
• Pre-/perinatal factors except maternal smoking not related to rolandic spikes.

## 1 Introduction

Developmental language disorder (DLD) is a term for childhood-onset language problems encompassing varying difficulties in both receptive and expressive language acquisition in multiple areas: phonology, semantics, word finding, morpho-syntax, pragmatics, language use, discourse, as well as verbal learning and memory (Bishop et al., 2017). The problems significantly affect children’s social interactions or educational progress and are not acquired or associated with a known biomedical cause (Bishop et al., 2017). DLD has long-term impacts: it is associated with lower academic and vocational qualifications in adulthood (Comti-Ramsden et al., 2018). The prevalence of DLD among kindergarten children is about 7.4% and a little higher among boys than girls (Tomblin et al. 1997). The etiology of DLD is still not fully understood. However, lower maternal education level, family history of DLD, later birth order, male gender, prematurity, presence of a newborn or a pregnancy condition, perinatal event, low 5-min Apgar score, maternal smoking or alcohol consumption during pregnancy were associated with an increased risk of DLD in a meta-analysis (Rudolph, 2017).

Landau-Kleffner syndrome (LKS) causes language regression (auditory agnosia) in children who have already acquired language skills, instead of the hindrance of language development as in DLD. It is a subtype of developmental and/or epileptic encephalopathies with spike-and-wave activation in sleep (EE-/DEE-SWAS), previously called epileptic encephalopathy with continuous spike-and-wave in sleep (CSWS), characterized by cognitive/behavioral regression, seizures, and marked epileptiform activation in EEG during sleep (Specchio et al., 2022). The EEG pattern (SWAS) was previously called electrical status epilepticus in sleep (ESES) and was defined as nearly constant (25–85%) epileptiform activity during NREM sleep (Sánchez Fernández et al., 2013). Antiepileptic drugs are used to treat EE-/DEE-SWAS to control epileptic seizures and ameliorate cognitive and/or behavioral regression (Sánchez Fernández et al., 2013). The fear of LKS is one of the main reasons why children with language problems are sent to EEG, even if they lack the main clinical feature of losing already acquired language skills.

Epileptiform activity or interictal epileptiform discharges (IEDs) or spikes are found most often in patients with epilepsy, representing the activity of the epileptic brain. However, incidental IEDs are also detected in 1.9–3.5% of healthy 1–15-year-old children (Cavazzuti et al., 1980, Eeg-Olofsson et al., 1971, Grant et al., 2016). Interictal epileptiform discharges are common in various neurological and developmental conditions, for example, broad cognitive development delay (47%, Asadi-Pooya, 2018), attention-deficit hyperactivity disorder (ADHD) (15%, Kartal et al., 2017), or autistic spectrum disorder (8–30%, Hrdlicka et al., 2004, Tuchman and Rapin, 1997).

Incidental IEDs are also common (6–94%) among children with DLD (Dlouha et al., 2020, Overvliet et al., 2010, Venkateswaran and Shevell, 2008), but there is a large variation in the IED prevalence estimates, partly because in many studies children have co-morbidities such as epilepsy. It is not known if IEDs in DLD children are associated with more severe language problems, although the abundant epileptiform activity is related to cognitive regression in CSWS (Sánchez Fernández et al., 2013). Consistent differences in EEG findings between varying subgroups (e.g., DLD with pronouncedly receptive and expressive difficulties) have not been found (Dlouha et al., 2020, Venkateswaran and Shevell, 2008).

There are some known risk factors for IEDs, according to one study: in the multiple regression analysis prematurity and perinatal asphyxia, and in the simple regression analysis, also maternal smoking during the prenatal period increased the risk, but 14% of the children in this study had a history of seizures (Kartal et al., 2017). To our knowledge, associations of pre-/perinatal factors with incidental IEDs have not been previously studied in children who do not have epileptic seizures.

In this study, we aimed to find out the prevalence of IEDs, if IEDs and other EEG findings are associated with the level of language performance, and if we find continuous spike-and-wave activation in sleep in these children who are neurologically healthy apart from DLD. Our secondary aim was to determine if pre- or perinatal factors are associated with the risk of IEDs.

## 2 Methods

### 2.1 Participants

Finnish 3–7-year-old children (n = 225) diagnosed with ICD-10 (WHO, 2010) as having DLD, participated in the Helsinki Longitudinal SLI Study (HelSLI) (Laasonen et al., 2018). The children had been referred to the Audiophoniatric Ward for Children, Department of Phoniatrics, Helsinki University Hospital for suspected DLD, and were examined during their evaluation period in the hospital ward. They had no sign of regression in cognitive or language development prior to the recruitment. They did not have any neurologic diseases (e.g., epilepsy or autism spectrum disorder), chromosomal abnormalities, intellectual disability (performance intelligence quotient (PIQ) below 70, Wechsler, 2003), hearing impairment, or oral anomalies. Finnish was their first or second language, and the children with Finnish as a second language had difficulties in both of their languages. The local ethics committee at Helsinki University Hospital approved the study protocol, and a written informed consent was obtained from the parents of the participating children.

### 2.2 Measures

#### 2.2.1 Language scores

Neuropsychologists and speech-language therapists examined the children in Finnish. Receptive Vocabulary subtest from WPPSI-III (Wechsler, 2003), Reynell Developmental Language Scales III, verbal comprehension scale (Edwards et al., 1997), and Receptive One-Word Picture Vocabulary Test 4 (Martin and Brownell, 2010a) were used to generate a receptive language score. Picture Naming subtest from WPPSI-III (Wechsler, 2003), the Boston Naming Test (Kaplan et al., 1983), the Reynell Developmental Language Scales III, Expressive Language Scale (Edwards et al., 1997), and the Expressive One-Word Picture Vocabulary Test (Martin and Brownell, 2010b) were used to generate an expressive language score. Information, Vocabulary, and Word Reasoning subtests from WPPSI-III (Wechsler, 2003), and Comprehension of Instructions from the Nepsy-II (Korkman et al., 2008) were used to generate a language reasoning score. We created sample standardized z-transformations of the tests mentioned above, and calculated their mean to create receptive, expressive, and language reasoning scores, respectively. The general language score was the mean of all three language scores. At least two of the measures of each score were needed for a score to be included in the analyses. Seven children (3%) were excluded due to this criterion.

#### 2.2.2 Eeg

Daytime routine EEGs with wake and sleep were recorded at the Department of Clinical Neurophysiology following clinical standards and using a 10–20 system and a NicoletOne device. If EEG had been recorded before the study onset, the recording was not done again. Seven children whose EEG was recorded more than five months prior to neuropsychological and speech-language therapy testing were excluded from these analyses. Thus, EEG was recorded max 0.92 months before or max 2.28 months after, and on average eight days after neuropsychological and language testing. We did not get EEGs from six children. The final number of children in the analyses was 205 (91%). The aim was to record a short wake episode and about 10 min of N2 sleep. To facilitate a nap, children were sleep-deprived, and melatonin could be administered. Epileptiform activity is usually particularly abundant at the beginning of NREM sleep (Nobili et al., 2001) and, therefore, also more abundant in a nap EEG than on average during the night (Larsson et al., 2010). Therefore, a nap EEG is a suitable test for the exclusion of SWAS/ESES, but to confirm the finding, an overnight EEG recording is recommended (Gardella et al., 2016, Gardella et al., 2019). Hyperventilation and a standard intermittent photic stimulation procedure were conducted during EEG. We recorded wake and stage N2-3 sleep in 200 (98%) and wake and stage N1 sleep in 4 (2%) EEGs. In one (0.5%) EEG, we did not manage to record sleep. The recordings were first evaluated for a clinical report by a clinical neurophysiologist on duty and re-evaluated for the study by one specialist in clinical neurophysiology (HRL) who consulted a senior specialist in clinical neurophysiology (LL) if there was a discrepancy between the initial evaluation and re-evaluation.

For the study, we evaluated the number and localization of ictal and interictal epileptiform discharges separately in wake and sleep, the presence of non-epileptiform background or focal abnormalities, and the presence of hypnagogic bursts with spiky morphology. The finding was considered as ESES/SWAS if epileptiform activity was more abundant in NREM sleep than in wake, was widespread, and present over 50% of the time in NREM sleep, obscuring normal sleep phenomena. Interictal epileptiform discharges were categorized as generalized or focal. Focal IEDs were categorized as frontocentrotemporoparietal (rolandic) if their maximum amplitude was in one of the following electrodes in an average reference montage: F4, F3, Fz, C4, C3, Cz, T4, T3, P4, P3, Pz and temporo-occipital if their maximum amplitude was in T6, T5, O2 or O1 electrodes. Focal IEDs with a maximum amplitude in Fp1 or Fp2 were not found.

#### 2.2.3 Potential IED risk factors

At the onset of the study, children’s parents filled in questionnaires concerning their child’s prenatal period and birth, general health and well-being, language and motor development level, as well as parents’ education level and developmental difficulties. Pre-/perinatal factors that had been associated with epilepsy/IEDs in previous studies were included in our analyses as potential risk factors for IEDs. We categorized gestation time as preterm (gestation under 37 weeks), full term (gestation 37–41 weeks), and post-term (gestation 42 weeks or more), and deliveries as “normal” or “complicated”. “Complicated” deliveries included, for example, vacuum extraction, elective or emergency cesarean section, labor induction, preeclampsia, or pre-labor rupture of membranes. We defined maternal smoking as no smoking or smoking (one or more cigarettes per day) during pregnancy.

#### 2.2.4 Potential confounding factors

Children’s age, gender, visuospatial intellectual ability (the Block Design from the WPPSI-III (Wechsler, 2003)), maternal education level (higher or primary/secondary education), and exposure to the Finnish language were considered as potential confounding factors. Children were classified as monolingual (Finnish) or bilingual, with one home language and Finnish as their second language. Bilingual children were divided to those who had been exposed to Finnish 1) for less than 24 months or 2) at least 24 months.

### 2.3 Data analysis

We used IBM SPSS Statistics 25 for statistical analyses. We first compared means of the general language score in children without IEDs and with 1) any, 2) frontocentrotemporoparietal (rolandic), 3) temporo-occipital, or 4) generalized IEDs and in children with and without 5) background or 6) focal EEG abnormalities, and 7) hypnagogic bursts using *t*-test. The same was done for the receptive, expressive, and language reasoning scores to see if the results differed from the general language score. According to the Shapiro-Wilk test, there was no significant deviation from the normal distribution (p = 0.142) in the general language score.

If an EEG variable was significantly associated with the mean of a language score according to the *t*-test, the associations of this EEG variable were further studied with logistic regression. If all four language scores had similar associations with an EEG variable, we did the logistic regression analyses only for the general language score, which was the mean of the other three language scores. We conducted three types of logistic regression models. The first models were simple logistic regression models with the EEG variable as the outcome and with one predictor variable. The language score, potential IED risk factors (gestation time, normal vs. complicated birth, maternal smoking during pregnancy), and potential confounding factors (visuospatial intellectual ability, exposure to Finnish language, maternal education level, age, gender) one at a time were used as predictor variables. In the second model, we tested if adjusting for age changed the results. Thus, all these second models included age plus one of the variables mentioned above, as predictors. In the third model (the final multiple regression model), we included the variables that were significantly (p < 0.05) associated with the EEG variable in the simple models and the confounding factors that were considered most meaningful for those variables.

## 3 Results

The age range of the children was 34.6–85.1 months (2.9–7.1 years). Other characteristics of the study population are shown in Table 1. There were no statistical differences between genders in potential confounding factors, language scores, EEG variables, or potential IED risk factors. The prevalence of any IEDs was 17%, of generalized IEDs 3.9%, and of focal IEDs 13%. Of the 26 children with focal IEDs, 6 (23%) had them only on the right, 4 (15%) only on the left, 7 (27%) only in the midline, 5 (19%) on both hemispheres, 2 (7.7%) on both hemispheres and in the midline, 2 (7.7%) on the left hemisphere and in the midline.

**Table 1:** Sample characteristics.

	Boys	Girls	All	p value (*t*-test / Chi-Square) for the difference between genders
n	158	47	205	
*Potential confounding factors*				
Age in months, mean (SD)	53.8 (10.6)	54.2 (9.84)	53.9 (10.4)	0.851
Visuospatial intellectual ability, mean (SD)	23 (4.1)	23 (3.1)	23 (3.9)	0.817
Maternal education				
• Primary or secondary, n (%)	87 (62)	24 (60)	111 (61)	
• Higher, n (%)	54 (38)	16 (40)	70 (39)	0.845
Monolingual, n (%)	94 (60)	28 (60)	122 (60)	
Bilingual, exposure to Finnish				
• 24 months or more, n (%)	34 (22)	12 (26)	46 (22)	
• less than 24 months, n (%)	30 (19)	7 (15)	37 (18)	0.742
*Language*				
General language score, mean (SD)	−0.047 (0.86)	0.089 (0.68)	−0.016 (0.82)	0.317
*EEG*				
Any EEG abnormality, n (%)	35 (22)	7 (15)	42 (21)	0.279
IEDs^a^ in EEG, n (%)	29 (18)	5 (11)	34 (17)	0.212
• frontocentrotemporoparietal (rolandic), n (%)	19 (13)	3 (6.7)	22 (11)	0.254
• temporo-occipital, n (%)	3 (2.3)	1 (2.3)	4 (2.3)	0.984
• generalized, n (%)	7 (5.1)	1 (2.3)	8 (4.5)	0.435
Abnormal background in EEG, n (%)	5 (3.2)	1 (2.1)	6 (2.9)	0.711
Focal slowing in EEG, n (%)	2 (1.3)	1 (2.1)	3 (1.5)	0.666
Hypnacogic bursts, n (%)	36 (23)	13 (28)	49 (24)	0.506
*Potential IED risk factors*				
Gestation under 37 weeks, n (%)	11 (7.1)	5 (11)	16 (7.9)	
Gestation 37–41 weeks, n (%)	126 (81)	39 (85)	165 (82)	
Gestation 42 weeks or more, n (%)	19 (12)	2 (4.3)	21 (10)	0.244
Complicated delivery, n (%)	53 (34)	14 (30)	67 (33)	0.693
Smoking during pregnancy, n (%)	28 (19)	8 (18)	36 (19)	0.912

a Interictal epileptiform discharges.

General, receptive, expressive, and language reasoning scores were significantly higher among children with rolandic IEDs than among children without IEDs. For other EEG variables (background abnormality, focal abnormality, hypnagogic bursts, any IEDs, temporo-occipital IEDs, generalized IEDs), there were no statistically significant differences in any of the language scores. The results for the general language score are shown in Table 2.

**Table 2:** EEG findings and general language score.

General language score (SD)			
	No	Yes	p value (*t*-test)^b^
IEDs^a^	−0.054 (0.82)	0.18 (0.82)	0.136
• frontocentrotemporoparietal (rolandic)		0.45 (0.78)	**0.007**
• temporo-occipital		−0.48 (0.57)	0.296
• generalized		−0.25 (0.70)	0.506
Abnormal background	−0.023 (0.82)	0.24 (0.89)	0.441
Focal slowing	−0.019 (0.82)	0.17 (0.50)	0.686
Hypnacogic bursts	−0.026 (0.80)	0.027 (0.88)	0.694

a Interictal epileptiform discharges.

b Significant p values in bold.

In simple logistic regression models for rolandic IEDs, increasing general language score, increasing age, and maternal smoking were associated with an increased risk of rolandic IEDs. Other factors did not have significant associations. Adjusting for age weakened the association of general language score with the risk of rolandic IEDs below the significance level. After adjusting for age, a new significant association was found only in bilinguals with the longest exposure time to Finnish.

Because age, general language score, and maternal smoking had a significant association with the risk of rolandic IEDs in the simple models, they were included in the final multiple logistic regression model as predictor variables.

Monolingual children had higher language scores than bilingual children because all children were tested in Finnish. Therefore, the exposure to the Finnish language was included in the final regression model as a potential confounding factor. We conducted logistic regression models also separately for monolingual and bilingual children, and the results were parallel to the results in the whole sample. However, group sizes became too small for making reliable conclusions in multiple regression analyses. Smoking was more common among mothers with primary/secondary education levels than among mothers with higher education. Therefore, maternal education level was also included in the final regression model as a potential confounding factor.

Thus, the variables in the final multiple regression model were: age, general language score, exposure to Finnish, and maternal education level. In this model, maternal smoking was associated with an increased risk of rolandic IEDs, while other variables did not have significant associations. The results from the simple, age-adjusted, and multiple logistic regression models are shown in Table 3. Table 4 shows the prevalence of IEDs in children whose mothers did or did not smoke during their pregnancy.

**Table 3:** Risk of frontocentrotemporoparietal (rolandic) IEDs^a^ in simple and multiple logistic regression models^b^.

	Simple		Adjusted for age		Multiple	
	OR	95% CI	OR	95% CI	OR	95% CI
*Potential confounding factors*						
Increasing age (in months)	**1.0**	**1.0**–**1.1**	–	–	1.0	0.95–1.1
Increasing visuospatial intellectual ability	1.0	0.94–1.2	0.91	0.77–1.1	–	–
Mother’s higher vs. primary/secondary education level	1.5	0.59–4.0	1.8	0.67–4.9	2.1	0.71–6.4
Monolingual vs. bilingual (exposure to Finnish less than 24 months)	0.96	0.30–3.1	0.91	0.27–3.0	3.2	0.48–21
Monolingual vs. bilingual (exposure to Finnish 24 months or more)	0.48	0.13–1.7	**0.23**	**0.056**–**0.92**	0.64	0.093–4.5
Gender (boy vs. girl)	2.1	0.58–7.3	2.1	0.58–7.5	–	–
*Language*						
Increasing general language score	**2.1**	**1.2**–**3.7**	1.8	0.97–3.4	2.5	0.88–7.0
*Potential IED risk factors*						
Gestation under 37 weeks vs. 38–41 weeks	1.1	0.23–5.1	0.97	0.20–4.7	–	–
Gestation 42 weeks or more vs. 38–41 weeks	0.84	0.18–3.9	0.75	0.16–3.6	–	–
Complicated delivery vs. normal delivery	0.71	0.29–1.8	1.6	0.64–4.2	–	–
Smoking vs. no smoking during pregnancy	**3.0**	**1.1**–**8.0**	**3.0**	**1.1**–**8.1**	**4.4**	**1.4**–**14**

a Interictal epileptiform discharges.

b Significant 95% confidence intervals in bold.

**Table 4:** Prevalence of IEDs^a^ among children by their motherś smoking status.

Smoking during pregnancy	No IEDs^a^, n (%)	Frontocentrotemporoparietal IEDs^a^, n (%)	Temporo-occipital IEDs^a^, n (%)	Generalized IEDs^a^, n (%)
No	133 (85)	13 (8.3)	4 (2.6)	6 (3.8)
Yes	27 (75)	8 (22)	0 (0.0)	1 (2.8)

a Interictal epileptiform discharges.

## 4 Discussion

We aimed to find out if IEDs and other EEG findings are associated with language performance and if we find electrical status epilepticus during slow-wave sleep (ESES)/spike-and-wave activation in sleep (SWAS) in this large and homogenous (co-morbidities excluded, age range relatively narrow) prospectively collected sample of neurologically healthy DLD children who were thoroughly studied (extensive neuropsychology and speech-language therapy testing and background information, EEG in wake and sleep). The language performance was not lower in children with EEG abnormalities, and we did not find ESES/SWAS in this population. The secondary aim was to determine if pre- or perinatal factors are associated with the risk of IEDs. An increased risk of incidental rolandic IEDs was related to maternal smoking during pregnancy. However, pre- or post-term delivery or complicated labor did not affect the risk of IEDs in these children.

Our sample was the largest one compared to the previous studies, but there was not even a trend toward lower language scores in children with IEDs. On the contrary, the general language score mean was higher (although not statistically significantly) in children with IEDs. The lack of associations between IEDs and language measures or perinatal risk factors has been attributed to small sample sizes (54–55 cases) in some previous studies (Dlouha et al., 2020, Parry-Fielder et al., 2009). Our sample of 205 children of whom 34 had and 171 had not IEDs, had 80% power with an α (two-tailed) of 0.05 to detect a difference of 0.433 in general language score (Kohn and Senyak, 2021). This was about half of the standard deviation and about 10% of the range of the general language score. Generalized and focal IEDs are however quite different in many aspects, and it is probable that the location of focal IEDs also makes a difference. Therefore, we wanted to study rolandic, temporo-occipital, and generalized IEDs separately. Surprisingly, when compared to children without IEDs, the language scores were statistically significantly higher (although adjusting for age weakened the association) in children with rolandic IEDs, and lower (not statistically significantly) in children with other IEDs. This indicates that different types of IEDs really should be analyzed separately. Unfortunately, the numbers were too low for reliably analyzing associations of temporo-occipital and generalized IEDs because they were uncommon in this population. Rolandic IEDs were more frequent, and we can reliably state that they were not associated with worse language scores, because the trend was contrary to that.

Incidental IEDs were relatively common (17%), and most of the IEDs were focal rolandic ones, but we did not find ESES/SWAS in this population. Thus, routine EEG recordings seem not to be necessary for children who have DLD but who do not have neurologic diseases, seizures, intellectual disability, or regression in cognitive development or behavior. The prevalence of IEDs among children with language problems has ranged from 2% (Nasr et al., 2001) to as high as 94% (Echenne et al., 1992) in previous studies. Variation is due to small sample sizes (32–111 children), differences in study populations, EEG recording methods (wake, daytime wake and sleep, vs. overnight), and diagnostic methods of DLD. Most studies are collected retrospectively. Some studies have included (Dlouha et al., 2020, Echenne et al., 1992, Mehta et al., 2015, Nasr et al., 2001, Tuchman et al., 1991,) while others have excluded (Parry-Fielder et al., 2009, Venkateswaran and Shevell, 2008) children with co-morbidities. Epilepsy and many other neurologic diseases and neuropsychiatric diagnoses increase the risk of IEDs (So, 2010).

Interpretation of physiologic EEG phenomena related to drowsiness and sleep, may also vary in this age group. Paroxysmal hypnagogic hypersynchrony with spike-like components is often encountered in EEGs of toddlers, and considered a benign finding, but it might be challenging to differentiate it from epileptiform discharges (Mizrahi, 1996). We found it in 24% of children, but it was not associated with language scores in our study. However, a recent study found an association between hypnagogic anterior predominant sharply contoured waveforms and increased risk of seizures and developmental delay (Datta, 2021).

The age range of the previous study samples has been broad, including typically both toddlers and school-aged children, and in some studies, even teenagers, which also affects results. Our results are in line with the study of Parry-Fielder et al., 2009 - the prevalence of IEDs being 13% in their study, vs. 17% in our study, and non-epileptiform abnormalities in their study 0%, vs. 4% in our study. Their sample was relatively small (54 cases, 45 controls) but the exclusion criteria, and recording and interpretation methods of EEG resemble ours. We lacked a control group, but this is like in most previous studies except the study of Parry-Fielder et al., 2009.

In previous studies, DLD children have usually been analyzed as one group or in some studies divided into varying subgroups (e.g., receptive and expressive DLD) without consistent differences in EEG findings between subgroups (Dlouha et al., 2020, Venkateswaran and Shevell, 2008). Based on our extensive neuropsychological and speech-language therapy test battery, we created language scores that acted as a measure of language reasoning, receptive and expressive language performance, and of general language performance. This enabled investigating if the severity or dimension of language impairment was associated with EEG findings. Significant associations were not found, but our analyses were cross-sectional. Therefore, it cannot be ruled out that EEG findings would still have a relationship with the prognosis of DLD in longitudinal analyses.

The risk of rolandic IEDs was not affected by pre-/post-term delivery or complicated labor in our study. It may be partly due to the study design, which excluded children with neurologic diseases that are known to associate with EEG abnormalities (So, 2010). Interestingly, the only significant risk found in our study was the increased risk (OR 4.4) of rolandic IEDs with maternal smoking during pregnancy. Kartal et al. (2017) also found this association, but since the sample size is relatively small, the significance of these findings needs to be confirmed.

## 5 Conclusions

To conclude, we showed that incidental IEDs are pretty common (17%) among 3–7 years old children with DLD, but not related to language performance cross-sectionally. Thus, routine EEGs do not bring additional information about language performance in this population. ESES/SWAS is not common in the DLD children who do not have neurologic diseases, seizures, intellectual disability, or regression of language development. Maternal smoking during pregnancy may increase the risk of incidental rolandic IEDs in offspring.

## Funding

This work was supported by Helsinki University; Kela [grants 407 and 20057]; and the Academy of Finland [grant 288435].

Funding organizations did not have a role in the study design, collection, analysis, or interpretation of the data, writing of the report, or in the decision to submit the article for publication.

## Author contributions

Hanna-Reetta Lajunen reviewed the EEGs, planned, and conducted the analyses, and wrote the manuscript. Pekka Lahti-Nuuttila took part in planning and conducting the analyses, generated the language scores, checked the data, and took part in writing the manuscript. Miika Leminen took part in planning the analyses and writing the manuscript. Sini Smolander and Sari Kunnari created some of the language variables, took part in planning the analyses and writing the manuscript. Eva Arkkila, Marja Laasonen, and Leena Lauronen planned the HelSLI-EEG research project, took part in planning the analyses in this study, and writing the manuscript. Marja Laasonen was the principal investigator of the whole HelSLI research project, of which HelSLI-EEG was a subproject. Leena Lauronen was responsible for the EEG recordings and took part in reviewing the EEGs. All authors have approved the final article.

## Declaration of Competing Interest

The authors declare that they have no known competing financial interests or personal relationships that could have appeared to influence the work reported in this paper.
